# The influence of the selection of macronutrients coupled with dietary energy density on the performance of broiler chickens

**DOI:** 10.1371/journal.pone.0185480

**Published:** 2017-10-20

**Authors:** Sonia Y. Liu, Peter V. Chrystal, Aaron J. Cowieson, Ha H. Truong, Amy F. Moss, Peter H. Selle

**Affiliations:** 1 Poultry Research Foundation, Sydney School of Veterinary Science, Faculty of Science, The University of Sydney, Camden, Australia; 2 School of Life and Environmental Sciences, Faculty of Science, The University of Sydney, Sydney, Australia; 3 Baiada Poultry Pty Limited, Pendle Hill, Australia; 4 DSM Nutritional Products, Kaiseraugst, Switzerland; 5 Poultry Cooperative Research Centre, University of New England, Armidale, Australia; National Institute for Agronomic Research, FRANCE

## Abstract

A total of 360 male Ross 308 broiler chickens were used in a feeding study to assess the influence of macronutrients and energy density on feed intakes from 10 to 31 days post-hatch. The study comprised ten dietary treatments from five dietary combinations and two feeding approaches: sequential and choice feeding. The study included eight experimental diets and each dietary combination was made from three experimental diets. Choice fed birds selected between three diets in separate feed trays at the same time; whereas the three diets were offered to sequentially fed birds on an alternate basis during the experimental period. There were no differences between starch and protein intakes between choice and sequentially fed birds (P > 0.05) when broiler chickens selected between diets with different starch, protein and lipid concentrations. When broiler chickens selected between diets with different starch and protein but similar lipid concentrations, both sequentially and choice fed birds selected similar ratios of starch and protein intake (P > 0.05). However, when broiler chickens selected from diets with different protein and lipid but similar starch concentrations, choice fed birds had higher lipid intake (129 versus 118 g/bird, P = 0.027) and selected diets with lower protein concentrations (258 versus 281 g/kg, P = 0.042) than birds offered sequential diet options. Choice fed birds had greater intakes of the high energy diet (1471 g/bird, P < 0.0001) than low energy (197 g/bird) or medium energy diets (663 g/bird) whilst broiler chickens were offered diets with different energy densities but high crude protein (300 g/kg) or digestible lysine (17.5 g/kg) concentrations. Choice fed birds had lower FCR (1.217 versus 1.327 g/g, P < 0.0001) and higher carcass yield (88.1 versus 87.3%, P = 0.012) than sequentially fed birds. This suggests that the dietary balance between protein and energy is essential for optimal feed conversion efficiency. The intake path of macronutrients from 10–31 days in choice and sequential feeding groups were plotted and compared with the null path if broiler chickens selected equal amounts of the three diets in the combination. Regardless of feeding regimen, the intake paths of starch and protein are very close to the null path; however, lipid and protein intake paths in choice fed birds are father from the null path than sequentially fed birds.

## Introduction

Modern broiler chickens have been strongly selected for superior growth performance including efficient feed conversion and breast-meat yield. The possibility for them to enhance performance by allowing birds to select ideal combinations of feedstuffs, has been demonstrated [1, 2]. Nutritional geometry relates feeding patterns and dietary nutritional properties to responses in growth performance and nutrient utilisation [3] and the determination of intake targets is essential to understand the nutritional priorities that govern feeding behaviour, diet selection and post-prandial metabolism in animal species [4]. Intake targets represent the quantity of nutrients that an animal requires to ingest in order to reach its nutritional target for growth and/or reproduction. Choice feeding is one means to quantify intake targets as choice feeding provides the opportunity for animals to select diets with different nutrient compositions. Hewson-Hughes, Hewson-Hughes [4] applied two approaches for diet selection; either sequential or choice feeding. Choice feeding provides animals with multiple diets to select at the same time and the objective is to identify intake targets by self-selection. In contrast, the same diets may be offered alternately under sequential feeding regimen to monitor the priorities of selection when animals are confronted with nutritionally unbalanced diets. The ability of animals to defend their nutrient intake targets when challenged in this manner becomes evident. The present study adopted both approaches to gain a better comprehension of macronutrient selection by broiler chickens.

Under nutritional geometry, the rules of compromise define the relative weighting given to the regulation of different nutrients when animals are constricted to unbalanced diets [5]. Liu, Selle [6] investigated the rule of compromise in macronutrient selection in broiler chickens and encountered the ‘equal distance rule’[3], which means animals consume feed to the point on their respective nutritional rails where the shortage of starch precisely equals the surplus of consumed protein. For optimal feed conversion efficiency, a balance between protein and energy, essentially derived from starch and lipid, is required [6]. Therefore, the objectives of this study were to investigate intake targets and preferences for macronutrients and energy densities by offering broiler chickens five dietary combinations with different concentrations of macronutrients under both sequential and choice feeding regimens.

## Materials and methods

### Diets preparation

Eight experimental diets were formulated based on maize, soybean meal, isolated soy protein, casein, synthetic amino acids, soybean oil and other minor ingredients to contain different levels of starch, protein, lipid and energy concentrations (Table 1). In Table 1, Experimental diet: S = starch, P = protein, L = lipid, E = energy. Capital, lowercase letters and letters with underline meant high, low and medium dietary concentration, respectively. For example, diet Spl contained high starch concentration and low protein and lipid concentrations; diet spl contained low lipid and medium starch and protein concentrations; diet PE contained high protein and high energy, respectively. All the experimental diets were formulated to similar ideal protein ratios with digestible lysine as the reference amino acid. The feeding study comprised a series of five dietary combinations to compare dietary selection under two feeding regimens (sequential or choice feeding). The details of dietary combination for choice feeding are listed in Table 2. Maize was hammer-milled through 3.2 mm sieve screen prior to mixing with other ingredients, which were then cold-pelleted and crumbled. Starch concentrations were determined by procedures based on dimethyl sulfoxide, α-amylase and amyloglucosidase[7]. Nitrogen concentrations were determined as outlined in Siriwan, Bryden [8]. Lipid concentration was determined by the automated Soxhlet extraction as described in Luque de Castro and Priego-Capote [9].

**Table 1:** Diet compositions, calculated and analysed nutrient specifications (g/kg) in experimental diets.

Ingredients/Diet	Spl^1^	spl	sPl	spl	spL	spl	Pe	PE
Maize	738	630	522	570	619	679	375	668
Soybean meal	207	238	270	250	230	218	503	36
Soya protein isolate	0.0	27.5	55.0	27.5	0.0	0.0	10.0	100
Casein	0.0	39.8	79.7	39.8	0.0	0.0	25.0	134
Soybean oil	0.30	0.15	0.00	20.2	40.4	20.4	0.00	0.00
Lysine HCl	1.20	0.73	0.25	0.53	0.80	1.00	0.50	0.00
Methionine	1.20	3.03	4.85	3.08	1.30	1.25	4.80	4.90
Threonine	0.20	2.45	4.70	2.40	0.10	0.15	1.20	8.20
Valine	0.00	0.15	0.30	0.15	0.00	0.00	0.60	0.00
Arginine	0.00	1.25	2.50	1.25	0.00	0.00	0.00	5.00
Salt	1.50	1.60	1.70	1.75	1.80	1.65	1.80	1.60
Sodium bicarbonate	2.60	1.83	1.05	1.68	2.30	2.45	2.10	0.00
Limestone	9.90	10.35	10.80	10.25	9.70	9.80	9.90	11.7
Dicalcium phosphate	11.50	9.75	8.00	9.85	11.70	11.60	8.00	8.00
Phytase^2^	0.10	0.10	0.10	0.10	0.10	0.10	0.10	0.10
Choline chloride 60%	0.40	0.40	0.40	0.40	0.40	0.40	0.40	0.40
Vitamin-mineral premix^3^	2.00	2.00	2.00	2.00	2.00	2.00	2.00	2.00
Cellulose	3.50	10.6	17.6	39.2	60.7	32.1	35.2	0.00
Celite^TM^	20.0	20.0	20.0	20.0	20.0	20.0	20.0	20.0
*Calculated*								
Metabolisable energy (MJ/kg)	12.75	12.67	12.58	12.67	12.75	12.75	11.04	14.12
Protein	160	230	300	230	160	160	300	300
Lipid	32	28	25	46	67	49	21	28
Starch	500	428	355	388	420	460	260	450
Calcium	8.7	8.7	8.7	8.7	8.7	8.7	8.7	8.7
Total phosphorus	5.3	5.3	5.3	5.2	5.2	5.3	5.9	4.6
Phytate P	2.4	2.4	2.4	2.3	2.2	2.3	3.0	1.8
Non-phytate P	3.0	3.0	2.9	3.0	3.0	3.0	2.9	2.9
Lysine^4^	8.5	13.0	17.5	13.0	8.5	8.5	17.5	17.5
Methionine	3.7	6.6	9.6	6.6	3.7	3.7	9.0	10.1
Methionine + cysteine	6.3	9.7	13.0	9.7	6.3	6.3	13.0	13.0
Threonine	5.6	10.2	14.8	10.2	5.6	5.6	11.6	17.9
Tryptophan	1.6	2.5	3.4	2.6	1.7	1.7	3.6	3.2
Arginine	9.4	14.0	18.6	14.2	9.8	9.6	19.0	18.2
Sodium	1.8	1.8	1.8	1.8	1.8	1.8	1.8	1.8
Potassium	7.7	8.1	8.5	8.1	7.7	7.7	12.5	4.4
Chloride	2.0	1.9	1.9	1.9	2.0	2.0	2.0	1.7
*Analysed* ^5^								
Starch	423	334	323	313	359	358	243	349
Protein	173	254	278	261	165	183	297	285
Lipid	25	26	23	39	59	46	22	29
Dry matter	858	859	864	865	861	861	859	863

^1^Experimental diet: S = starch, P = protein, L = lipid, E = energy. Capital, lowercase letters and letters with underline meant high, low and medium dietary concentration, respectively. For example, diet Spl contained high starch concentration and low protein and lipid concentrations. Diet PE contained high protein and high energy, respectively.

^2^Axtra PHY TPT was supplemented at 1000 FTU/kg

^3^The vitamin-mineral premix supplied per tonne of feed: [MIU] retinol 12, cholecalciferol 5, [g] tocopherol 50, menadione 3, thiamine 3, riboflavin 9, pyridoxine 5, cobalamin 0.025, niacin 50, pantothenate 18, folate 2, biotin 0.2, copper 20, iron 40, manganese 110, cobalt 0.25, iodine 1, molybdenum 2, zinc 90, selenium 0.3

^4^Digestible basis

^5^As-is basis and protein was analysed as N x 6.25; lipid was analysed by Soxhelt extraction

**Table 2:** Analysed macronutrient concentrations of diets.

Dietary combinations		Diet	Energy (MJ/kg)	Starch (g/kg)	Protein (g/kg)	Lipid (g/kg)
1	Fixed energy	Spl^1^	12.75	423	173	25
		sPl	12.58	323	278	23
		spL	12.75	359	165	59
2	Fixed lipid and energy	Spl	12.75	423	173	25
		spl	12.67	334	254	26
		sPl	12.58	323	278	23
3	Fixed starch and energy	sPl	12.58	323	278	23
		spl	12.67	313	261	39
		spL	12.75	359	165	59
4	Fixed protein and energy	Spl	12.75	423	173	25
		spl	12.75	358	183	46
		spL	12.75	359	165	59
5	Fixed protein	Pe	11.04	243	297	22
	Different energy	sPl	12.58	323	278	23
		PE	14.12	349	285	29

^1^Experimental diet: S = starch, P = protein, L = lipid, E = energy. Capital, lowercase letters and letters with underline meant high, low and medium dietary concentration, respectively. For example, diet Spl contained high starch concentration and low protein and lipid concentrations. Diet PE contained high protein and high energy, respectively.

### Bird management

This feeding study complied with the specific guidelines approved by the Animal Ethics Committee of the University of Sydney (Project No. 601). Male day-old chicks (Ross 308) were received from a commercial hatchery and were offered a commercial starter diet to 9 days post-hatch. They were then identified (wing-tags), weighed and allocated into bioassay cages on the basis of body weight in an environmentally-controlled facility. There was no statistical difference between the average body weight for each cage at the beginning of the feeding study. A factorial design was used with five combinations of three feeds (or diets) being fed either sequentially or as a choice. This resulted in ten treatments which were replicated six times using six chicks per replication. A total of 360 chicks were offered experimental diets from 10 to 31 days post-hatch. Broilers had unlimited access to water and feed under a ‘23-hour-on-1-hour-off’ lighting regime for the first three days, and then under a ‘16-hour-on-8-hour-off’ lighting regime for the remainder of the study. The room temperature was maintained at 32°C for the first week, then gradually decreased to 22 ± 1°C by the end of the third week and maintained at the same temperature until the end of the feeding study. Body weight and feed intake were recorded from which feed conversion ratios (FCR) were calculated. The incidence of dead or culled birds was recorded daily and their body-weight was used to adjust FCR calculations. Nutrient intakes in the final mixture that broiler chickens selected were calculated from analysed macronutrient concentrations and intakes of each diet.

The experiment included two feeding regimens: sequential and choice feeding, as described in Hewson-Hughes, Hewson-Hughes [4]. For choice feeding, broiler chickens were offered three diets in three separate feed trays simultaneously. To avoid any positional bias, the position of each feed tray was rotated on a daily basis. For sequential feeding, broiler chickens were cycled through three 6-day periods followed by one 3-day period. From 10–28 days post-hatch, broiler chickens were offered different diets on every two of the six days. From 29–31 days post-hatch, broiler chickens were offered different diets on each of the three days. There are six possible orders for the presentation of three diets to broiler chickens, in order to reduce sequence effects, broiler chickens were randomly assigned to one of the six orders of diet presentation in the entire experimental feeding period.

In dietary combination 1, broiler chickens were offered three diets (Spl, sPl and spL) with variable dietary starch, protein and lipid concentrations but similar metabolisable energy (~ 12.69 MJ/kg). In dietary combination 2, diets Spl, spl and sPl with fixed lipid concentrations (~ 25 g/kg) and energy density (~ 12.63 MJ/kg) but variable starch and protein concentrations were offered to broiler chickens. In dietary combination 3, diets sPl, spl and spL with fixed starch concentrations (~ 332 g/kg) and energy density (~ 12.67 MJ/kg) but variable protein and lipid concentrations were offered to broiler chickens. In dietary combination 4, diets Spl, spl and spL with fixed low protein concentrations (~ 174 g/kg) and energy density (~ 12.75 MJ/kg) but variable starch and lipid concentrations were offered to broiler chickens. In dietary combination 5, diets Pe, sPl and PE with fixed high protein concentrations (~ 287 g/kg) and variable metabolisable energy density were offered to broiler chickens. As described above, in each dietary combination, the three diets were provided to broiler chickens sequentially or as choice.

### Sample collection and chemical analysis

Total excreta were collected at 25–27 days post-hatch from choice fed birds to determine parameters of nutrient utilisation, which included apparent metabolisable energy (AME), AME to gross energy ratios (AME:GE), nitrogen (N) retention and N-corrected apparent metabolisable energy (AMEn). Feed intake of each diet in the choice feeding combination was recorded during this period to calculate intakes of energy and nitrogen. Excreta were air-forced oven dried for 24 h at 80°C. The gross energy (GE) of diets and excreta were determined by bomb calorimetry using an adiabatic calorimeter (Parr 1281 bomb calorimeter, Parr Instruments Co., Moline, IL). Nitrogen contents of diets and excreta were determined using a N determinator (Leco Corporation, St Joseph, MI, USA).

In the case of diet combinations 1 and 5, two birds whose body weight was close to the cage mean were selected for analysing carcass composition. Birds were euthanized by intravenous injection of sodium pentobarbitone. The carcass was weighed with feathers but without organs to calculate carcass yield before processing. The carcass was subsequently autoclaved, ground and freeze-dried to analyse for GE, N and lipid concentrations, as described previously.

### Calculations

The AME values of the diets were calculated on a dry matter basis from the following equation [10]:

$${AME}_{diet}=\frac{\left(Feed\;intake\times {GE}_{diet})-(Excreta\;output\;\times {GE}_{excreta}\right)}{\left(Feed\;intake\right)}$$

AME:GE Ratios were calculated by dividing AME by the GE of the appropriate diets. N contents of diets and excreta were determined using a nitrogen determinator (Leco Corporation, St Joseph, MI) and N retention was calculated from the following equation [8]:

$$Retention\;(\%)=\frac{\left(Feed\;intake\;\times {Nutrient}_{diet})-(Excreta\;output\;\times {Nutrient}_{excreta}\right)}{\left(Feed\;intake\times {Nutrient}_{diet}\right)}\times 100$$

N-corrected AME (AMEn MJ/kg DM) values were calculated by correcting N retention to zero using the factor of 36.54 kJ/g N retained in the body [11].

Intake path was plotted by recording feed intake of individual diets every six days in choice fed birds and every two days in sequentially fed birds. By comparing intake path of choice and sequentially fed birds and this investigated animal’s regulatory capacity of prioritising nutrient intakes when nutrient imbalanced diets were provided. The intake of nutrients was calculated from the following equation,

Nutrientintake=Feedintake×Dietarynutrientconcentration

### Statistical analysis

**Fig 1:**
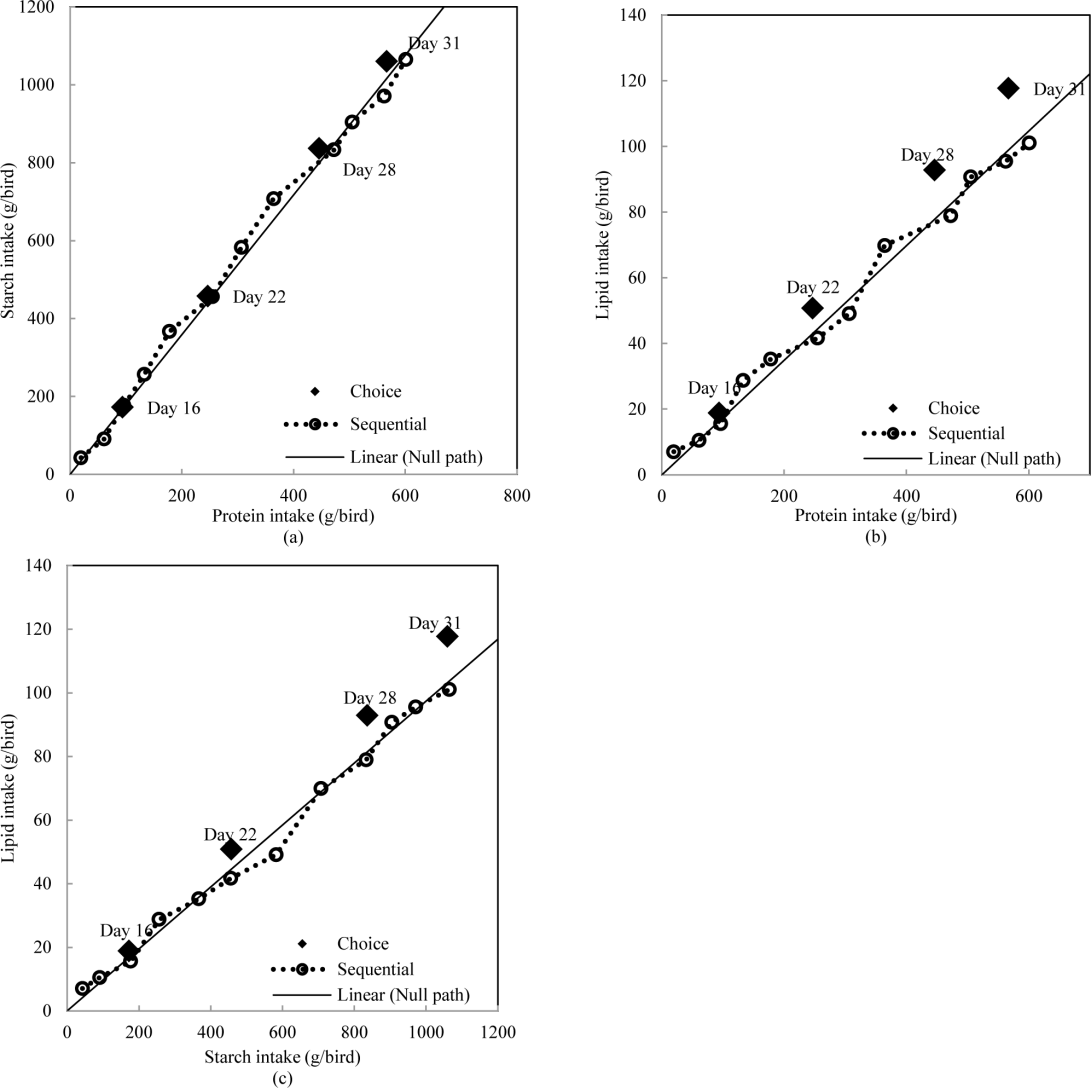
Intake paths of macronutrients in broiler chickens offered diets with different concentrations of starch, protein and lipid.

**Fig 2:**
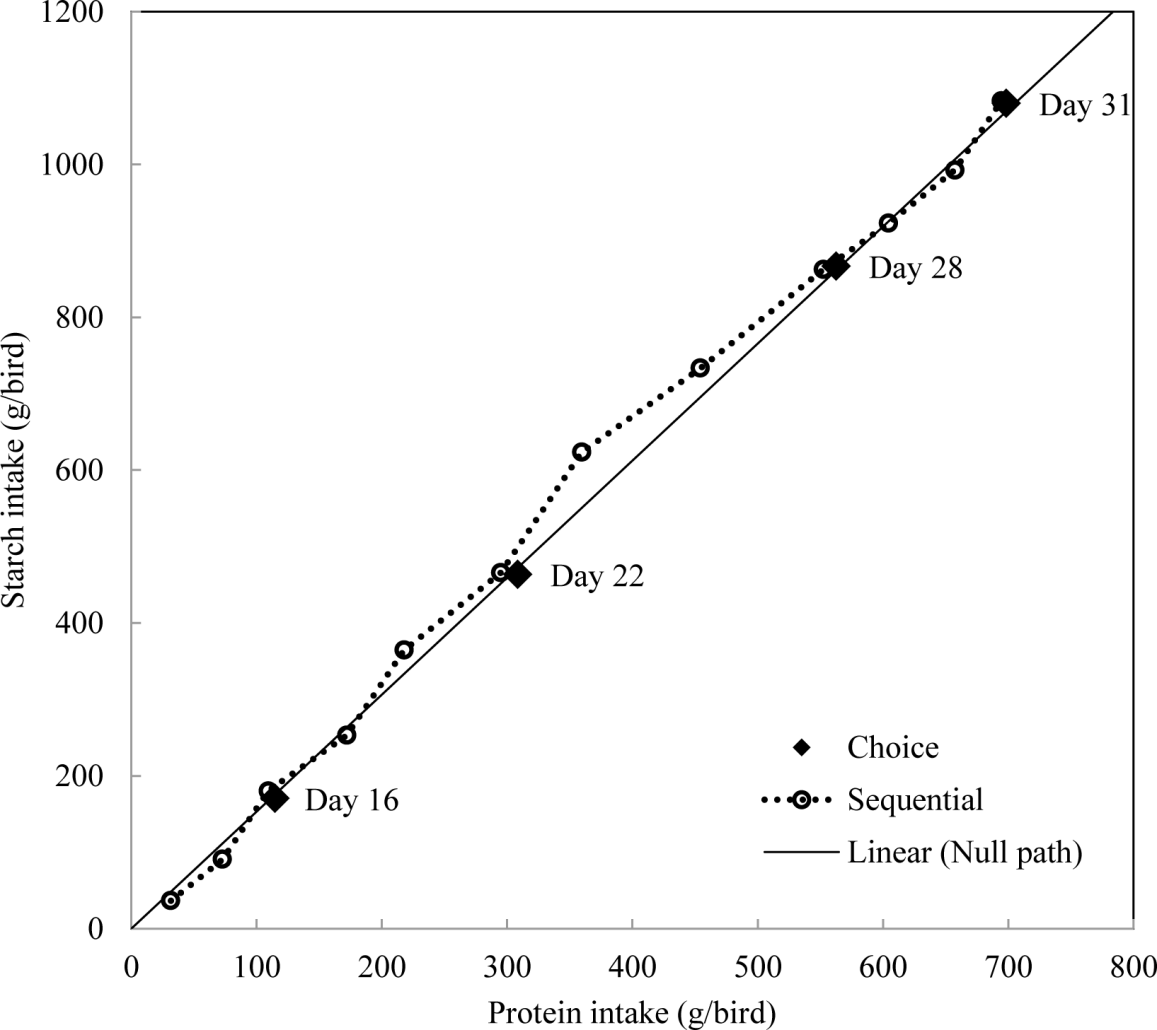
Intake paths of protein and starch in broiler chickens offered diets with different starch and protein concentrations.

**Fig 3:**
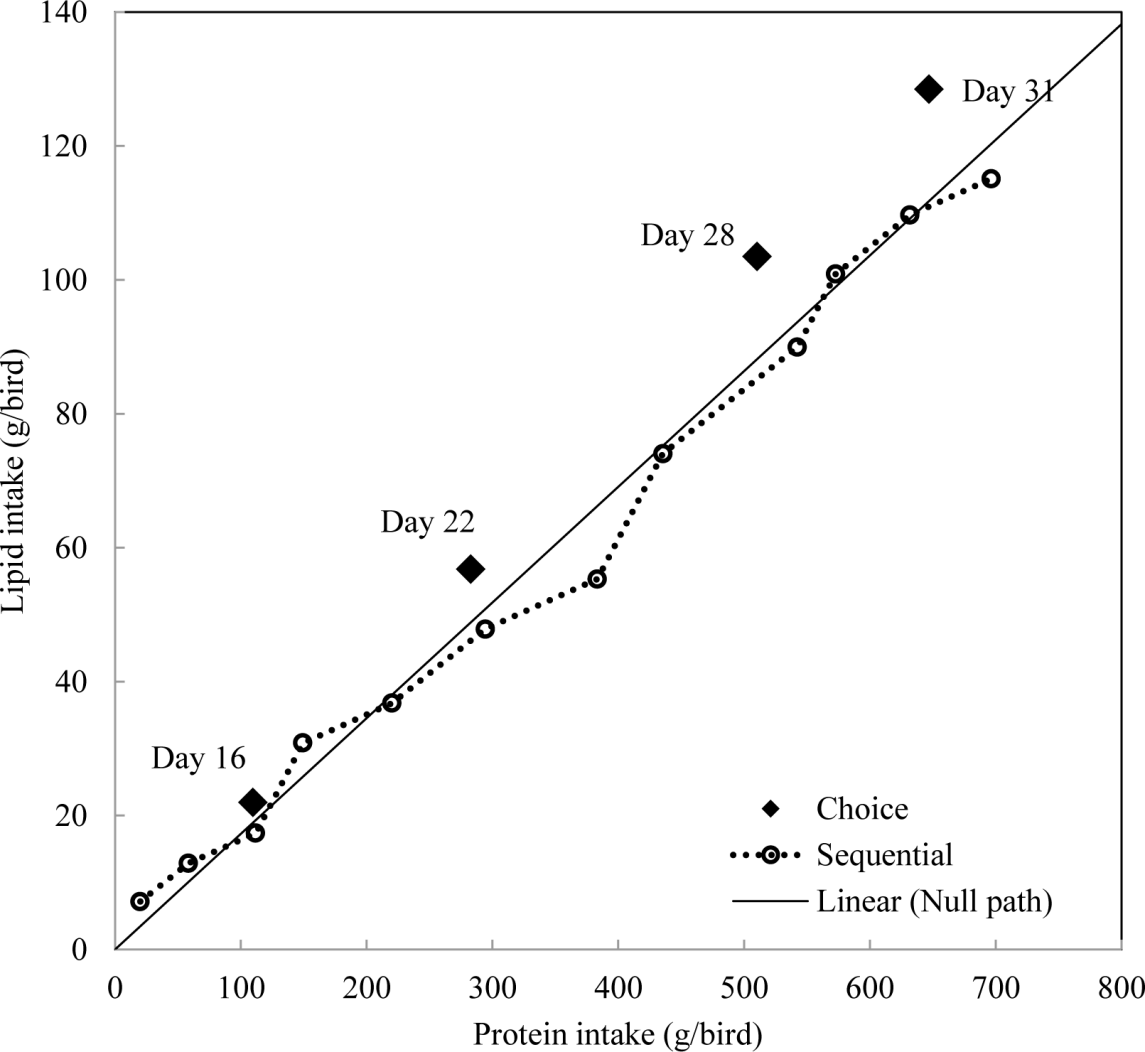
Intake paths of protein and lipid in broiler chickens offered diets with different protein and lipid concentrations.

Experimental data were analysed using JMP® 9.0.0 (SAS Institute Inc. JMP Software. Cary, NC). The experimental units were replicate cage means and statistical procedures included analyses of variance using general linear models and a probability level of less than 5% was considered to be statistically significant. The experiment was conducted by Completely Randomized Design with a 2 x 5 factorial arrangement which were replicated six times using six chicks per replication. The factors were feeding regimens and diet combinations (Table 3). For each dietary combination, intakes of single experimental diets and nutrients between two choice feeding approaches were compared by pair-wise comparison (Tables 4 and 5). Only birds with choice feeding regimen were selected for determination of nutrient utilisation in Table 6 and they were analysed by Completely Randomized Design with single factor (5 dietary combinations). Only birds offered dietary combination 1 and 5 were harvested for carcass traits determination, pair-wise comparison was conducted to compare the influence of feeding regimens. The Null Hypothesis in both methods of presenting feed to broiler chickens is that the birds will consume 33.3% of each feed; therefore, in Table 4, percentage intakes of each diet were compared against 33.3% by One-Sample t-Test to determine significance. Accumulated nutrient intakes were used to plot Figs 1–3 and the solid line represents the intake path if broiler chickens selected equal amount of each diet.

**Table 3:** The influence of feeding regimens and diet combinations on growth performance and mortality rate in broiler chickens from 10–31 days post-hatch.

Diet combination		Feeding regimen	FI	WG	FCR	Mortality rate
			(g/bird)	(g/bird)	(g/g)	(%)
1	Spl, sPl, spL	Sequential	2501	1692	1.480^c^^d^	0.00^b^
2	Spl, spl, sPl	Sequential	2626	1871	1.404^d^^e^	2.78^b^
3	sPl, spl, spL	Sequential	2522	1880	1.342^e^	0.00^b^
4	Spl, spl, spL	Sequential	2413	1411	1.710^b^	0.00^b^
5	Pe, sPl, PE	Sequential	2433	1836	1.327^e^	0.00^b^
1	Spl, sPl, spL	Choice	2485	1649	1.515^c^	0.00^b^
2	Spl, spl, sPl	Choice	2596	1879	1.394^d^^e^	2.78^b^
3	sPl, spl, spL	Choice	2566	1780	1.443^c^^d^	2.78^b^
4	Spl, spl, spL	Choice	2522	1333	1.898^a^	0.00^b^
5	Pe, sPl, PE	Choice	2331	1915	1.217^f^	11.11^a^
	SEM		43.29	42.69	0.0331	1.884
	*Main effects*:*Feeding regimen*					
	Sequential		2499	1738	1.453	0.56
	Choice		2500	1711	1.493	3.33
	*Diets*					
1	Spl, sPl, spL		2493^b^	1670^b^	1.497	0.00
2	Spl, spl, sPl		2611^a^	1875^a^	1.399	2.78
3	sPl, spl, spL		2544^a^^b^	1830^a^	1.392	1.39
4	Spl, spl, spL		2467^b^^c^	1372^c^	1.804	0.00
5	Pe, sPl, PE		2382^c^	1876^a^	1.272	5.56
	*P-Value*					
	Diets		0.982	0.325	0.058	0.024
	Feeding regimen		<0.0001	<0.0001	<0.0001	0.025
	Interaction		0.164	0.241	<0.001	0.019

^abcdef ^Means within a column not sharing common superscripts are significantly different

**Table 4:** The influence of feeding regimens and diet combinations on the total intakes of each diet in broiler chickens from 10–31 days post-hatch.

Dietary combination	1			2			3			4			5		
Feeding regimen	Diet	Intake (g/bird)	Intake (%)	Diet	Intake (g/bird)	Intake (%)	Diet	Intake (g/bird)	Intake (%)	Diet	Intake (g/bird)	Intake (%)	Diet	Intake (g/bird)	Intake (%)
Sequential	Spl	858^b^	34.3	Spl	939	35.8**	sPl	878^b^	34.8*	Spl	802^b^^c^	33.2	sPl	880^b^	36.2***
	sPl	868^b^	34.7	spl	922	35.1*	spl	909^b^	36.0**	spL	864^b^	35.8***	Pe	829^b^	34.0
	spL	775^b^	31.0**	sPl	766	29.2***	spL	735^c^	29.1***	spl	747^c^^d^	31.0***	PE	725^c^	29.8***
Choice	Spl	673^c^	26.9***	Spl	865	33.3	sPl	703^c^	27.4**	Spl	718^d^	28.4**	sPl	663^c^	28.3*
	sPl	639^c^	25.8**	spl	955	36.8*	spl	695^c^	27.1***	spL	1080^a^	42.9***	Pe	197^d^	8.4***
	spL	1173^a^	47.3***	sPl	775	29.9**	spL	1168^a^	45.5**	spl	723^d^	28.7**	PE	1471^a^	63.2***
*SEM*		32.7			33			32			26.8			28	
Main Effects: Feeding regime															
Sequential		834			875			841			804			811	
Choice		828			865			855			841			777	
Diet	Spl	765		Spl	902^a^		sPl	791		Spl	760		sPl	771	
	sPl	754		spl	938^a^		spl	802		spL	972		Pe	513	
	spL	974		sPl	770^b^		spL	951		spl	735		PE	1098	
P-value															
Feeding regimen		0.844			0.706			0.585			0.107			0.145	
Diet		<0.0001			<0.0001			<0.0001			<0.0001			<0.0001	
Interaction		<0.0001			0.249			<0.0001			<0.0001			<0.0001	

^abcd ^Means within a column not sharing common superscripts are significantly different

* percentage intake is significantly different from 33.3% (P < 0.05)

** P < 0.01

*** P < 0.001

**Table 5:** The influence of feeding regimens on the total intakes^1^ of macronutrients and energy in the final mixture chosen by broiler chickens from 10–31 days post-hatch.

Feeding regimen	Nutrient intake (g/bird)			Nutrient composition (g/kg)			Energy intake	Energy density
*Combination 1* *(Spl*, *sPl*, *spL)*	Starch	Protein	Lipid	Starch	Protein	Lipid	(MJ/bird)	(MJ/kg)
Sequential	1079	605	103^b^	436	244	57^b^	32.0	12.93
Choice	1061	571	115^a^	424	228	66^a^	31.6	12.63
SEM	26.5	13.2	2.1	12.3	6.5	0.8	0.72	0.348
P-Value	0.648	0.100	0.002	0.517	0.107	<0.0001	0.938	0.556
***Combination 2 (Spl*,** ***sp******l*, *sPl)***								
Sequential	1116	716	76	431	277	29	33.6	12.95
Choice	1098	713	75	419	272	29	33.2	12.64
SEM	14.3	9.5	1.0	12.7	8.1	0.9	0.42	0.234
P-Value	0.377	0.871	0.565	0.491	0.704	0.570	0.539	0.376
***Combination 3(sPl*, *s******pl*****, *spL)***								
Sequential	982	706	118^b^	391	281^a^	47	32.5	12.66
Choice	1003	665	129^a^	390	258^b^	50	32.6	12.96
SEM	21.2	14.6	3.2	11.8	6.9	1.8	0.69	0.321
P-Value	0.492	0.078	0.027	0.962	0.042	0.192	0.881	0.533
***Combination 4 (Spl*,** ***s******p******l*****, *spL)***								
Sequential	1071	488	123	425	194	49	30.9	12.26
Choice	1084	496	130	450	206	54	31.5	13.09
SEM	30.0	14.2	4.0	13.5	6.2	1.7	0.89	0.393
P-Value	0.763	0.695	0.261	0.209	0.181	0.059	0.641	0.165
***Combination 5 (Pe*, *sPl*, *PE)***								
Sequential	866	817	60^b^	392^a^	369^a^	27	30.8	13.21
Choice	894	779	68^a^	349^b^	304^b^	27	31.3	12.88
SEM	17.7	15.3	1.4	9.7	8.8	0.7	0.62	0.291
P-Value	0.228	0.110	0.002	0.011	<0.001	0.586	0.569	0.437

^ab ^Means within a column not sharing common superscripts are significantly different

^1^dry matter basis

## Results

The average weight gain and FCR for all experimental treatments from 10 to 31 days post-hatch was 1724 g/bird and 1.473 g/g, respectively, which were 9.5% higher than the weight gain (1575 g/bird) and 3.7% more efficient than the FCR (1.550 g/g) stipulated in 2014 Ross 308 performance objectives. The influence of feeding regimens and diet combinations on growth performance and mortality rate is shown in Table 3 (S1 and S2 Tables). The influence of feeding regimens and diet combinations on total intakes of each diet, macronutrients and energy in broiler chickens is included in Tables 4 and 5 (S3 and S4 Tables).

### Dietary combination 1: Variable starch, protein and lipid

There were no differences in feed intake, weight gain, FCR or mortality rate when diets Spl, sPl and spL were offered to broilers either sequentially or as choice (Table 3). However, there were interactions in both absolute and percentage intakes of each diet between feeding regime and diet type (P < 0.0001). For absolute intakes, there were no differences in diet intakes in broiler chickens fed sequentially, whereas, choice fed birds consumed significantly (P< 0.0001) more diet spL than either diet Spl or sPl (Table 4). For percentage intakes, birds offered diet spL had a significantly lower (P < 0.0001) intake than 33.3% in sequential feeding groups (Table 4); however, it had significantly higher percentage intake than 33.3% in choice feeding groups. No difference in energy intakes was observed (P = 0.938, Table 5).

### Dietary combination 2: Variable starch and protein

Broilers were given a choice between starch and protein and they were offered diets Spl, spl and sPl. There were no significant differences between sequential and choice feeding groups on feed intake, weight gain, FCR and mortality rate from 10–31 days post-hatch (P > 0.50, Table 3). However, regardless of feeding regimen, broiler chickens consumed significantly (P < 0.0001, Table 4) less diet sPl (770 g/bird, 29.5%) than diet Spl (902 g/bird, 34.6%) and spl (938 g/bird, 35.9%). There were no significant differences in macronutrient intakes between the sequential and choice feeding regimen (P > 0.35, Table 5). On average, broiler chickens chose diet combinations containing 425 g/kg starch, 275 g/kg protein and 29 g/kg lipid. No difference in energy intakes was observed (P = 0.539, Table 5).

### Dietary combination 3: Variable protein and lipid

Broilers were given a choice between protein and lipid and they were offered diets sPl, spl and spL. Broiler chickens offered diets as choice had significantly lower weight gain (1780 versus 1880 g/bird, P = 0.015) and FCR (1.443 versus 1.342 g/g, P = 0.002) than sequential feeding birds from 10–31 days post-hatch (Table 3). There were no significant differences in feed intake and mortality rate between broiler chickens under different feeding regimen (P > 0.30). There were interactions in both absolute intakes of each diet between feeding regimen and diet type (P < 0.0001, Table 4). Broiler chickens in choice feeding group consumed more diet spL (1168 g/bird, 45.5%, P < 0.0001) than diets sPl or spl. However, diet spL had significantly lower (P < 0.0001) intake than diet sPl or spl in sequentially fed birds. For nutrient intakes, choice fed broiler chickens had significantly higher absolute (129 versus 118 g/bird, P = 0.027) lipid intake and selected significantly lower protein concentration (258 versus 281 g/kg, P = 0.042) than birds offered diet options sequentially. Sequentially fed birds chose diet combination of 391 g/kg starch, 281 g/kg protein and 47 g/kg lipid, whereas, choice fed birds selected diet combination of 390 g/kg starch, 258 g/kg protein and 50 g/kg lipid (Table 5). No difference in energy intakes were observed between different feeding groups (P = 0.881).

### Dietary combination 4: Variable starch and lipid

Broilers were given a choice between starch and lipid and they were offered diets Spl, spL and spl. There were no differences in feed intake, weight gain and mortality rate between sequential and choice feeding groups. However, broiler chickens offered diets as choice had significantly higher FCR (1.898 versus 1.710 g/g, P = 0.004) than birds offered diets sequentially (Table 3). There were interactions in absolute intakes of individual diet between feeding regimen and diet type (P < 0.0001, Table 4). Broiler chickens had statistically higher than 33.3% intake of diet spL under both feeding regimens (P < 0.001). There were no significant (P > 0.05, Table 5) differences in nutrient and energy intakes between sequentially and choice fed birds, except lipid concentration, which tended to be significantly higher in choice fed birds (54 versus 49 g/kg, P = 0.059). On average, broiler chickens selected diet combination of 438 g/kg starch, 200 g/kg protein and 52 g/kg lipid.

### Dietary combination 5: Variable energy

The balance between protein and energy was considered in dietary combination 5 where diets sPl, Pe and PE were offered to broiler chickens. There were no differences in weight gain between sequentially and choice fed birds (P = 0.127). Broiler chickens offered diet options had significantly lower feed intake (2331 versus 2433 g/bird, P = 0.03) and FCR (1.217 versus 1.327 g/g, P < 0.0001) than sequentially fed birds (Table 3). However, choice fed birds also had significantly higher mortality rate (11.11 versus 0.00%, P = 0.01) than sequentially fed birds. There were interactions in absolute intakes of individual diet between feeding regimen and diet type (P < 0.0001). Choice fed broiler chickens consumed more Diet PE (1471 g/bird, 63.2%, P < 0.0001) than diets sPl or Pe (Table 4). For nutrient intakes, choice fed broiler chickens had significantly higher lipid intake (68 versus 60 g/bird, P = 0.002) than birds offered diet options sequentially. Choice fed birds also selected significantly lower starch (P = 0.011) and protein (P < 0.001) concentrations. Sequentially fed birds consumed a diet containing 43 g more starch, 65 g more protein and the same amount of lipid as choice fed broilers (Table 5). No difference in total energy intakes were observed between different feeding groups (P = 0.569).

### Selected measurements of nutrient utilisations and carcass traits

The influence of dietary combinations on nutrient utilisation in choice feeding groups from 27–29 days post-hatch is shown in Table 6 (S5 Table). There was no significant difference in excreta moisture between different dietary combinations (P = 0.373). Combination 4 had the highest AME, N retention and AMEn (P < 0.0001). By comparison, Combination 2 had the lowest AME and AMEn and Combination 5 had the lowest N retention. The influence of feeding regimen on carcass traits of broiler chickens at 30 days post-hatch in Combination 1 and 5 is shown in Table 7 (S6 and S7 Tables). In Combination 1, there were no significant differences between different feeding regimen on carcass weight, yield, carcass protein and gross energy. In contrast, in Combination 5, choice feeding groups had a significantly higher carcass weight (1944 versus 1802 g/bird, P = 0.032) and carcass yield (88.1 versus 87.3%, P = 0.012).

**Table 6:** Nutrient utilisations in choice fed birds from 27–29 days post-hatch.

Dietary combination	AME (MJ/kg)	N retention (%)	AMEn (MJ/kg)	Excreta moisture (%)
1 *(Spl*, *sPl*, *spL)*	15.62^b^	72.58^b^	13.68^b^	67.67
2 *(Spl*, *sp**l*, *sPl)*	14.97^c^	67.68^c^^d^	12.87^d^	66.54
3 *(sPl*, *s**pl*, *spL)*	15.11^c^	70.59^b^^c^	12.98^d^	67.42
4 *(Spl*, *s**p**l*, *spL)*	16.08^a^	77.03^a^	14.29^a^	68.71
5 *(Pe*, *sPl*, *PE)*	15.52^b^	66.52^d^	13.31^c^	63.03
SEM	0.084	1.105	0.092	2.064
P-value	<0.0001	<0.0001	<0.0001	0.373

^abcd ^Means within a column not sharing common superscripts are significantly different (P < 0.05)

**Table 7:** The influence of feeding regimens on carcass traits at 30 days post-hatch in Dietary combination 1 and 5.

Dietary combination	Feeding regimen	Carcass weight (g/bird)	Carcass yield (%)	Carcass^1^ protein (%)	Carcass GE^1^ (MJ/kg)
1 *(Spl*, *sPl*, *spL)*	Sequential	1638	86.3	56.2	26.3
	Choice	1614	86.5	56.8	25.8
	*SEM*	65.7	0.26	1.08	0.36
	*P-Value*	0.798	0.642	0.681	0.342
5 *(Pe*, *sPl*, *PE)*	Sequential	1802^b^	87.3^b^	64.3	23.7
	Choice	1944^a^	88.1^a^	66.3	23.4
	*SEM*	40.4	0.19	1.21	0.24
	*P-Value*	0.032	0.012	0.270	0.453

^1^dry matter basis

## Discussion

### Defending lipid intake target

It is important to understand the regulatory capacities of multiple nutrient intakes in animals and voluntary nutrient intake targets when chickens are given free access to all dietary options. However, when broiler chickens were restricted to a single unbalanced diet, the priority of nutrient intake regulation was declared. Dietary combination 1 explored the preference of starch, protein and lipid in broiler chickens offered iso-energetic diets. Fig 1 illustrated the intake path of macronutrients in choice and sequential feeding groups when broiler chickens were offered dietary combination Spl, sPl and spL from 10–31 days post-hatch. When starch intake was plotted against protein (Fig 1A), choice fed birds selected a constant dietary starch to protein ratios of 1.88 g/g, which was derived from significant linear correlations between starch and protein intake (y = 1.880x–4.788, R^2^ = 0.99). Sequentially fed birds selected similar dietary starch to protein ratios at the end of each feeding cycle. The solid line in Fig 1 represents the null path if broiler chickens consumed equal amount of the three diets. The intake paths of choice and sequentially fed birds are close to the null path. Similar outcomes are evident in Fig 2 which compared intake paths of protein and starch in broiler chickens offered diet combination Spl, spl and sPl as sequential and choice. Both intake paths reached almost the same destination at 31 days post-hatch. This suggested that broiler chickens were able to defend starch versus protein intake targets when they were restricted to one diet at a time. This is consistent with the ‘rule of compromise’ reported in Liu, Selle [6], where the ‘equal distance rule’ was observed and broiler chickens consumed feed to the point on its respective nutritional rails where the shortage of starch exactly equals the surplus of consumed protein.

For dietary combination 1, sequentially fed birds had significantly lower lipid intakes (103 versus 115 g/bird, P = 0.002) and lipid concentrations (57 versus 66 g/kg, P < 0.0001) than choice fed birds. This suggested that lipid intake target was more difficult to defend than starch and protein. Fig 1B and 1C compared intakes of lipid with protein and starch. Choice fed birds generated intake path which is away from the null path and sequentially fed birds had intake path close to the null path. Choice fed birds selected a dietary lipid to protein ratio of 0.209 g/g (y = 0.209x–0.805, R^2^ = 0.99, P < 0.0001) and a dietary lipid to starch ratio of 0.111 g/g (y = 0.111x–0.272, R^2^ = 0.99, P < 0.0001). However, sequential feeding birds selected dietary lipid to protein ratio of 0.167 g/g (y = 0.167x + 2.602, R^2^ = 0.99, P < 0.0001) and dietary lipid to starch ratio of 0.095 g/g (y = 0.095x + 0.841, R^2^ = 0.99, P < 0.0001). Sequentially fed birds failed to maintain similar dietary nutrient ratios, especially when broiler chickens were older than 16 days post-hatch. Similar outcomes were observed in Fig 3 which compared intake paths of protein and lipid in broiler chickens offered diet combination sPl, spl and spL as sequential and choice where choice fed birds had significantly higher lipid intake than sequentially fed birds (129 versus 118 g/bird, P = 0.027). Choice fed birds selected dietary lipid to protein ratio of 0.199 g/g (y = 0.199x + 0.411, R^2^ = 0.99, P < 0.0001), whereas sequential feeding birds selected dietary lower lipid to protein ratio of 0.165 g/g (y = 0.165x + 1.618, R^2^ = 0.99, P < 0.0001).

One selection criterion for modern broiler chickens is appetite to ensure maximal growth rates and feed intakes increase during grower phase as birds mature. From 16 days post-hatch, a two-day interval of diet rotation was of sufficient length to prevent broiler chickens from readjusting their intakes for lipid. Conversely, when three diets were available at the same time for broiler chickens to select, they had higher lipid intakes than sequentially fed birds. Moreover, there were no differences (P > 0.50) in energy intakes during the experimental period between sequentially and choice fed birds (32.0 versus 31.6 MJ/bird in Dietary combination 1; 32.5 versus 32.6 MJ/bird in Dietary combination 3; 30.8 versus 31.3 MJ/bird in Dietary combination 5). This may be because lipid possesses the highest energy density amongst standard feed ingredients [12] which would facilitate dietary adjustments. Moreover, Noblet, Milgen [13] indicated that lipid (31.5 MJ/kg) generates higher net energy densities than starch (14.4 MJ/kg) and protein (10.2 MJ/kg) in pigs. In choice fed birds in Dietary combination 1, broiler chickens selected 47.3% of diet spL which was higher in lipid than the other two diets. Diet spL contained the highest lipid concentration (59 g/kg) than diet Spl (29 g/kg) and diet sPl (23 g/kg). Similar findings were observed in Dietary combinations 3–5 where diets with the highest lipid concentration were consumed in greater amount in choice feeding groups. Klasing [14] suggested that addition of lipid to low fat diets increases feed intake in chickens offered iso-energetic diets, and this phenomenon may be due to palatability. It has been argued that birds have a lower taste acuity compared to mammals due to their low taste bud numbers; however, chickens are able to quickly adapt their feeding behaviour based on taste cues and the ratio of the number of taste buds/oral cavity volume is higher than in most mammals [15]. Mabayo, Okumura [16] fed chickens flavoured or non-flavoured diets with medium-chain and long-chain triacylglycerol and found that birds showed a significant preference for non-flavored diets over flavored diets. This suggests that gustation may play a role in determining feed intake in chickens.

### Diets with low protein concentrations

It is noteworthy that there was obvious feed wastage in Dietary combination 4 caused by feed flicking. Feed wastage was not observed in other diet combinations. However, feed wastage was not quantifiable, thus feed intake calculations for broiler chickens offered diets Spl, spL and spl were biased by feed wastage. This may have contributed to the highest FCR (1.804 g/g) and feed flicking may be associated with the low amino acid concentrations in the three diets. The formulated concentrations of lysine, sulphur amino acids and threonine were 8.5, 6.3 and 5.6 g/kg, respectively. Low amino acid concentrations may have contributed to less efficient feed conversion in broiler chickens in Dietary combination 4. This is supported by previous studies Liu, Selle [6] and Liu, Selle [17] where both reported that FCR was decreased with increasing dietary protein concentrations. Similar findings were observed in Jackson, Summers [18] and Collin, Malheiros [19]. Jackson, Summers [18] showed when protein concentrations were increased from 160 to 360 g/kg, FCR was significantly improved from 2.29 to 2.11 g/g in broiler chickens from 0–49 days post-hatch. Collin, Malheiros [19] showed a low-protein diet (126 g/kg) had a FCR of 2.82 g/g compared to average FCR of 1.71 g/g in a normal protein diet (197 g/kg) in broiler chickens from 0–42 days post-hatch. Moreover, broilers retained significantly less energy as protein in low-protein diets than the average retained protein energy in diets with normal protein concentrations (123 versus 285 kJ/kg BW^0.75^ per day, P < 0.0001). A meta-analysis based on seven studies using sorghum-based diets as a dietary model suggested that FCR was correlated with both protein and starch digestion rates, but protein digestion rate was more important than starch to feed efficiency and muscle protein deposition [20]. Therefore, it was expected that diets with lower amino acid or crude protein concentrations had suboptimal FCR.

Different from growth performance, broiler chickens offered low protein diet combination in Dietary combination 4 had significantly (P < 0.0001) better N retention (77.03%), AME (16.08 MJ/kg) and AMEn (14.29 MJ/kg) than broiler chickens offered other diet combinations. Consistently, Jackson, Summers [21] reported that increasing dietary protein levels from 160 to 360 g/kg reduced protein utilisation by 48.4% from 34.9 to 18.0% and reduced energy utilisation by 17.1% from 27.5 to 22.8%. The authors concluded that protein and energy utilisation was negatively correlated with protein intake. Similar observations were made in Liu, Selle [6], where N retention was negatively correlated with dietary protein to starch ratios (P < 0.01).

Choice fed birds had significantly higher FCR (1.898 versus 1.710 g/g) than sequentially fed birds in Dietary combination 4. The lack of differences in nutrient intakes between choice and sequentially fed birds indicated the variation in FCR between two feeding groups was more likely due to nutrient utilisation (Table 6). Also, choice fed birds had liberty to select from three diets while birds with sequentially fed birds were restricted to one diet only at any one time. Therefore, diet selection in birds with choice feeding may have caused a higher energy expense on feeding compared to birds with sequential feeding. Nir, Hillel [22] suggested that lack of uniform particle size in mash diets reduces feed efficiency because of the increased time and energy for chicks to select larger particles.

### Protein and energy balance

Liu, Selle [6] showed that protein is more important than non-protein components in the diets for muscle protein deposition. Liu, Selle [17] reported quadratic relationships between FCR and protein:AME ratios in the diet and FCR was improved with increasing protein:AME ratios until predicted minimal FCR of 1.066 g/g was reached when protein:AME ratio equalled 19.32. Liu and Selle [20] considered the balance of starch and protein digestion and its relevance to feed efficiency as both digestion of protein and starch and absorption of amino acids and glucose are required for muscle protein deposition. In Dietary combination 5, in order to determine the importance of the balance of protein and energy, all three diets were formulated to contain high amino acid concentrations, including lysine (17.5 g/kg), sulphur amino acids (13.0 g/kg) and threonine (11.6 to 17.9 g/kg) but their energy density varied from 11.04 to 14.12 MJ/kg. Emmans [23] suggested that birds eat to satisfy their requirement for the most limiting nutrient in the ration; thus non-protein energy could be limiting in high protein diets. Indeed, broiler chickens chose to eat 63.2% diet PE which contained the highest energy density, and this led to a higher mortality rate (11.11 versus 0.00%, P = 0.01) in choice feeding groups. Consistently, Liu, Selle [17] also reported significantly higher mortality in diets containing high protein, high lipid and low starch, which had an efficient feed conversion of 1.146 g/g but an excessive mortality rate of 37.5%. The higher mortality rate may be due to the metabolic stress of high nutrient intakes and rapid growth in high yielding, modern broiler chickens.

In Dietary combination 5, choice fed birds had 8.3% significantly lower FCR than those offered diets sequentially (1.217 versus 1.327, P < 0.0001). Sequentially fed birds selected higher starch (392 versus 349 g/kg, P = 0.011), protein (369 versus 304 g/kg, P < 0.001) and energy (13.21 versus 12.88 MJ/kg, P = 0.437) concentrations than birds with choice feeding (Table 5) and they had higher FCR and lower weight gain. Sequentially fed birds had restricted access to a balanced diet, which may cause asynchrony between protein and non-protein energy. For example, sequentially fed birds consumed 829 g/bird diet PE; in contrast, choice fed birds only consumed 197 g/bird diet Pe (P < 0.0001, Table 4). Broiler chickens were forced to eat diet PE under the sequential feeding regimes as only one diet was available at a time, and diet PE contained similar protein (digestible lysine) concentrations to diet sPl and PE but lower starch and lipid concentration. During the experimental period, choice fed birds had significantly higher lipid intake than sequentially fed birds (68 versus 60 g/bird, P = 0.002). In addition, the major source of protein in experimental diets was soybean meal, casein and isolated soy protein. In contrast to diet sPl and Pe, diet PE had the highest inclusion of casein and isolated soy protein and this may have contributed to better FCR in choice fed birds. Liu and Selle [20] suggested the rate of protein digestion is more important to feed conversion efficiency than starch and rapidly digestible protein is beneficial to feed efficiency and muscle protein deposition. Both casein and isolated soy protein are considered to be sources of rapidly digestible protein and higher consumption of diet PE in choice feeding group may have contributed to significantly better feed conversion efficiency and higher carcass weight (1944 versus 1802 g/bird, P = 0.032).

## Conclusions and implications

The present study predictably demonstrated suboptimal growth performance in diets containing low-protein concentrations, especially as poor feed conversion efficiency. The balance of protein and energy is essential for optimal feed conversion and carcass yields. When broiler chickens were offered diets with different metabolisable energy but the same high amino acid concentrations, they were able to select the diet with the highest energy. This led to the significantly lower FCR and higher carcass weights and yields in choice feeding groups than sequential feeding. In combinations with diets containing different dietary lipid concentrations, broiler chickens had lower lipid intake under sequential feeding regimes and this suggested that broilers were not able to defend their lipid intakes under a diet rotation interval of two days. In addition, the diet containing the highest lipid concentration supported the highest feed intakes in choice fed birds. This may be due to the high energy density of lipid or the lipid “taste” in the diet. Relatively less attention has been paid to lipid than starch and protein in poultry nutrition research; however, lipid has the potential to closely regulate feed intake and it is an expensive energy component in poultry diets. Therefore, future studies exploring the interaction between two major energy components–lipid and starch in diets with different energy density are required to confirm the impact of lipid on feed intake and other growth performance parameters.

## Supporting information

S1 Table Growth performance in all the cages. (XLSX)

S2 Table Individual bird weight and weight gain calculation in each cage. (XLSX)

S3 Table Feed intake calculation in sequentially fed birds. (XLSX)

S4 Table Feed intake calculation in choice fed birds. (XLSX)

S5 Table Calculation of nutrietn utilisation in each cage. (XLSX)

S6 Table Carcass weight of individual bird in each cage. (XLSX)

S7 Table Carcass composition of individual bird in each cage. (XLSX)
